# Biobank donation in search of public benefits and the potential impact of intellectual property rights over access to health-technologies developed: A focus on the bioethical implications

**DOI:** 10.1093/medlaw/fwae010

**Published:** 2024-04-24

**Authors:** Aisling M McMahon, Opeyemi I Kolawole

**Affiliations:** School of Law and Criminology, Maynooth University, Maynooth, Co. Kildare, Ireland; School of Law and Criminology, Maynooth University, Maynooth, Co. Kildare, Ireland

**Keywords:** Biobank, Bioethics, Informed Consent, Intellectual Property

## Abstract

The availability of biomaterials is a key component of health research and the development of new health-technologies (including, diagnostics, medicines, and vaccines). People are often encouraged by biobanks to donate samples altruistically to such biobanks. While empirical evidence suggests many donors are motivated by the desire to contribute towards developing new health-technologies for society. However, a tension can arise as health-technologies whose development is contributed to by donors’ biomaterials will often be protected by intellectual property rights (IPRs), including patents. Patents give rightsholders control over how patented technologies are used and can be used in a way that impedes public access to technologies developed. Yet, there are no binding European legal obligations mandating disclosure to donors of how IPRs can operate over downstream health-technologies and how they could impact access to health-technologies developed, nor are there legally binding obligations to ensure public accessibility of technologies developed. Focusing on the bioethical implications posed, this article argues that the current situation can impact donors’ autonomy and dignity interests. A more holistic approach is needed for biobank donation, which embeds a consideration of donors’ expectations/interests from the point of donation through to how such samples are used and how health-technologies developed are accessed. We put forward avenues that seek to address such issues.

## I. INTRODUCTION

The availability of human biomaterials is a key component of health research which can prove critical to our understanding of health, disease, and the development of downstream therapeutics, vaccines, and diagnostics (hereafter ‘health-technologies’).^1^ To obtain biomaterials, scientific studies could recruit participants to donate samples (and related data) directly for a study or obtain biomaterials from biobanks. A biobank is: ‘[a] collection of biological material and the associated data and information stored in an organised system, for a population or a large subset of a population’.^2^ Different types of biobanks exist, from large-scale population-level biobanks,^3^ to disease-specific and specialised biobanks, such as those focusing on the storage of particular types of biological material (eg the UK Stem Cell Bank).^4^ Biobanks are a key resource providing biological samples to a community of researchers.^5^

However, a tension can arise because the pathways encouraging people to donate samples to publicly funded biobanks—and often a key motivation for donors in donating—tend to focus on donors’ ability and desire to help others under an altruistic framework, including by contributing to developing new health-technologies for the public.^6^ Yet, downstream health-technologies that are developed—and whose development is contributed to by knowledge from these biomaterials—will often be protected by intellectual property rights (IPRs), including patent rights.^7^ As discussed below, such IPRs tend to vest primarily in the scientific inventor(s) or their employer(s),^8^ with no link to donors/biobanks. Rightsholders generally do not have legally binding obligations to consider donors’ interests or expectations in how they use such IPRs, and IPRs may be used in a way that hinders the public’s access to health-technologies.

Under the current publicly funded biobank model, there is a clear separation or bifurcation between the donation stage of the biobank process at which point we see a focus on public interests/benefits that donors can contribute to, and the second stage in the biobanking process involving the use of samples. In this usage stage, in Europe, there is no set or uniform model agreement used by biobanks and third-party researchers who use biobank samples, and biobanks are not legally mandated to include specific provisions in agreements between them and third-party users to mandate or encourage reasonable public access to the downstream technologies developed. Instead, the terms of such agreements are generally at the discretion of the biobank and third parties entering into such agreements. While those who donate biospecimens to biobanks generally have limited, if any, control over any products or knowledge derived from their samples after donation.^9^ If technologies are developed using - or are contributed to by knowledge derived from the use of - biobank samples, IPRs over such technologies are controlled by the relevant rightsholders whose intentions may not align with the donors’ altruistic intentions.^10^

Building upon existing work which examines IPRs that may arise in the biobank context – including related to downstream research using biobank samples – more generally,^11^ but focusing here specifically on the bioethical interests at stake in such contexts, this article argues that the current legal framework within Europe governing: (i) how IPRs that may arise over downstream technologies are communicated with biobank donors in the donation phase, and (ii) how such downstream IPRs may be used in a manner that can impact public access to such technologies, gives rise to significant potential bioethical implications. There has been limited scrutiny of such bioethical issues to date, and this article seeks to fill this gap. We make two key arguments: First, we argue that the lack of binding legal obligations for biobanks to disclose to donors the potential for future IPRs over health-technologies developed *and* how these may impact access to health-technologies can impact donors’ *autonomy*. Secondly, we argue that this lack of a disclosure obligation combined with the lack of legal obligations to provide avenues to intervene over how IPRs can be used by rightsholders—if unreasonable public access impediments arise—could lead donors to feel their donation of biomaterials (and related data) was a means to an end, thereby potentially impacting their dignity interests. Such issues stem from the bifurcation of the biobank donation and usage phases. We argue that a more holistic approach is needed for publicly funded biobanks where donors’ (and public) interests around the potential use of future IPRs are considered from the donation phase through to the usage phase, and we put forward several avenues to achieve this.

Due to space limitations, this article focuses on biobanking; however, such arguments have broader resonance for other contexts where individuals participate in and provide biomaterials (and related data) for health research in other contexts, including clinical trials. Importantly, this article is not arguing that IPRs should not arise over downstream health-technologies whose development is contributed to by biobank samples—under the current health innovation model, we recognise the need for a balance between the incentives IPRs can provide for the development of new health-technologies and the potential impacts IPRs can have on access to technologies. We are not arguing that biobank donors should obtain IPRs over downstream technologies developed. One of the authors has examined this issue in more detail elsewhere.^12^ While it is not reopened here, for the purposes of the argument, the key points are summarised (Section III) below.

Finally, it is important to highlight that this article does not seek to contribute to discussions critiquing the operation of the applicable IPR systems in the health innovation context per se.^13^ Instead, our contribution here focuses on the current context, within which we are interested in the statements made to donors to encourage them to donate samples to publicly funded biobanks (and donors’ motivations around donating), which often centre around the potential for donors to contribute to the development of better understandings of health/disease and the development of new health-technologies for the public benefit. The article is interested in how such statements/motivations sit with the reality of how any benefits from research using donor’s samples, if developed (including new health-technologies), can be accessed by the public. This article’s core focus—developed further below—is around how the operation of IPRs over downstream benefits arising within the current publicly funded biobank systems can give rise to a potential tension with donors’ expectation of contributing to publicly accessible benefits and how this potential tension can be mediated *within the confines of the current system.*

The article is structured as follows: Section II outlines empirical work demonstrating that altruistic motivations around contributing to the public interest and developing new health-technologies are key motivations for many donors to donate to biobanks, and that biobanks often use altruistic framings in encouraging people to donate samples to biobanks. Section III illustrates that downstream health-technologies developed using such samples – or knowledge derived from these – can be protected by IPRs and outlines how such IPRs can impact access to such health-technologies. It highlights that there are no binding legal obligations in Europe mandating: (i) biobanks to disclose to donors the potential for IPRs to arise over downstream health-technologies developed and how these can impact access to such technologies; (ii) nor are there binding legal obligations related to how IPRs over such technologies can be used. Section IV makes the case that the lack of such legal obligations—gives rise to bioethical implications, including potentially impacting donors’ autonomy and dignity interests. Section V argues that a more holistic approach is needed for how IPRs are dealt with in the biobank context and proposes avenues to ameliorate bioethical issues arising. Section VI concludes, arguing that greater scrutiny is needed over the bioethical implications posed by how potential IPRs arising from knowledge/developments contributed to by biobanks are communicated with donors/public and how such IPRs are used downstream, within the biobanking (and broader health research) context.

## II. DONATION TO BIOBANKS AND THE PROMISE OF PUBLIC BENEFITS

Various empirical studies identify altruistic motivations to contribute to health/society as a key factor in people’s decisions to donate samples to biobanks.^14^ Domaradzki and Pawlikowski,^15^ in their review of empirical studies examining public attitudes to biobank donation, highlighted the following key motivations for donating to biobanks: ‘a general feeling of duty^16^ and a desire to contribute to the common good,^17^ ... helping others^18^ and future generations.^19^ Many wished to help generate new knowledge and develop new therapies.^20^ Others expected benefits to their families, relatives or ethnic groups,^21^ or desired medical service and research results’.^22^

For example, Kettis-Lindblad and others’ study involved members of the Swedish public who were asked whether they would donate blood samples for research. Of the potential donors who expressed a willingness to donate, their main motivations included a desire to ‘benefit future patients (89%), benefit for myself or my family’ (61%)’.^23^ Some (32.1%) were motivated by ‘a sense of duty’.^24^ Although we acknowledge that not all donors donate based on public interest motivations, these studies indicate that the potential for public benefits to arise from such donations appears to be a key factor for many donors in participating in biobanking.

Similarly, a focus on the public interest and health benefits often underpins how publicly funded biobanks frame donors’ participation and the use of biomaterials in the biobank.^25^ There tends to be an emphasis on a promise of potential public benefits through donation, underpinning publicly funded biobank models.^26^ For example, the UK Biobank states that:

UK Biobank is a large-scale biomedical database and research resource … *It is a major contributor to the advancement of modern medicine and treatment and has enabled several scientific discoveries that improve human health.*^27^ [Emphasis added]

Similarly, the UK Parkinson’s Brain Bank states that:

By studying brain tissue from people with and without Parkinson’s we are beginning to understand why these nerve cells die. This is vital to developing treatments that can slow, stop or even reverse Parkinson’s.

Studying donated brain tissue has already led to major advances in our understanding of Parkinson’s – including identifying changes in the brain cells affected in the condition, such as increased levels of iron.

As a result, new treatments are currently being developed … .^28^

A focus on using biobank samples to generate new knowledge and understandings for health contexts is also evident in the National Irish COVID Biobank, which states that ‘your participation will allow researchers to address many key questions in COVID-19 research and we are grateful for your time and donation of your biological samples and healthcare data’.^29^ Similarly, at a regional level, the ‘Biobanking and BioMolecular resources Research Infrastructure – European Research Infrastructure Consortium’ (BBMRI-ERIC), a European research infrastructure for biobanking, states that: ‘[u]ltimately, our goal is to make new treatments possible’.^30^ Such statements highlight a focus on the health benefits to which donors can contribute to. Here, we are not questioning the potential for biobank samples/data to contribute to the development of new understandings and health-technologies. Instead, we refer to such statements to demonstrate that biobanks often emphasise the importance of donated samples for developing new understandings and technologies/treatments in the health context, and contributing to such benefits can be a key factor motivating some donors to contribute biomaterials to biobanks. Despite this, as we now consider, there is limited discussion in the donation phase around how public access to new technologies (including new treatments) developed could be potentially impeded by IPRs.

## III. BIOBANKS AND DOWNSTREAM HEALTH-TECHNOLOGIES: IPRs POTENTIAL IMPACTS ON ACCESS TO HEALTH-TECHNOLOGIES

New health-technologies developed using or whose development is contributed to by biomaterials donated to a biobank(s) will likely be protectable by IPRs. A range of IPRs can apply to such downstream technologies, including trade-secret protection on know-how or production methods for such technologies, and patents over elements of a product such as a vaccine, medicine, or relevant process.^31^ As patent rights are one of the most common types of IPRs that may arise over downstream health-technologies arising in such contexts, we focus on patents here as an exemplar of issues that can arise. However, notably, a producer/manufacturer may strategically layer different types of IPRs over health-technologies, to increase the scope of its legal rights and interests over a technology. For example, for vaccines, rightsholders may use trade secret protection for aspects of know-how that are difficult to replicate, such as how to make certain elements of a vaccine, and patent other elements of a vaccine that may be more straightforward to reverse engineer.^32^

A patent allows rightsholders to exclude others from commercially exploiting—without the rightsholder’s permission (license)—a ‘technology’ for the term of the patent right (generally 20 years).^33^ Within World Trade Organisation (WTO) member States, which includes 164 States worldwide, patents must be made available in all fields of technology, including health-technologies. Some exclusions to patentability apply,^34^ but there are no exclusions from patentability due to a technology being developed using altruistically donated biomaterials.

Under the patent system, patents are granted to the scientific inventor(s) of a patentable ‘technology’ or their employer(s).^35^ No legal provisions give donors of biobank samples a right to claim patent rights that may arise over technologies developed by researchers using—or contributed to by—donors’ samples.^36^ Under European patent law, biobanks do not gain IPRs over technologies developed using such samples. Instead, any health-technologies developed following the use of samples drawn from a publicly funded biobank may be protected by patents (and other IPRs) held by the researcher (or their employer) who invented that technology. Any control donors have is prescribed in legal agreements donors sign on donating material to the biobank, which should also be outlined in the informed consent agreements with the biobank. Generally, such agreements provide no ongoing rights for donors to control the use of knowledge/products generated using samples.^37^

We are not arguing that those who donate samples to the biobank should obtain a share of patents arising over downstream technologies in such contexts. As one of us has discussed elsewhere,^38^ this would likely neither be feasible nor practically desirable for various reasons, including: (i) samples from multiple donors may be involved in developing a technology. It may be difficult to ascertain which donors should benefit; (ii) it could create multiple obligations which may limit incentives to use biobank samples; (iii) knowledge gleaned from samples is usually combined with other knowledge to develop a technology, so it is questionable to what extent donors should share any patents that may arise; and (iv) relatedly, the patent system rewards the intellectual endeavour involved in the development of new technical inventions. Donated biomaterials could be construed as being more akin to raw materials in the development process. Thus, for these reasons and others, this article does not argue that donors share in such patents or any commercial benefits accruing to IPR rightsholders per se. Nonetheless, how downstream IPRs may be used and how these IPRs may potentially impede public access to such technologies is a crucial consideration that warrants deeper scrutiny.

Turning to how patents may impact access and use of downstream technologies. Patents allow rightsholders to control certain aspects of who uses a patented technology (for commercial purposes) for the patent duration and on what terms.^39^ If a third party uses a patented technology for commercial purposes without rightsholder(s) permission (license), they could—in certain circumstances—be challenged on the basis of patent infringement. How rightsholder(s) use patents and other IPRs can impact who can access patented technologies, including health-technologies such as a medicine. For example, rightsholders may choose to price a patented medicine at a level which exceeds what many in the population or the national government can afford. Rightsholders may also refuse licenses to third parties to produce a patented medicine. This may decrease the supply of that medicine, which can incentivise or enable rightsholders to charge a premium price for the medicine, given the likely demand.

For many technologies, multiple IPRs may apply over different elements, multiple rightsholders may hold these IPRs, and, as noted, rightsholders may strategically layer the IPRs that apply. Rightsholders may also apply for patents on new uses of technology towards the end of the initial patent term of 20 years (so-called evergreening).^40^ These are broader health innovation issues which are not unique to biobanking. However, such issues, when combined, mean that for many biobanks, there can be a dissonance between the promise of public benefits in the biobank donation phase and how IPRs can be used in a manner that may limit public access to downstream health-technologies that may be developed in the usage phase.

In effect, it is plausible that medicines treating a condition may be developed using knowledge derived from biomaterials donated to a biobank. However, IPRs over such medicines may be used in a way that contributes to such technologies being unaffordable to the public or a section of the public who needs them, including donors or their families.^41^ Moreover, in the NHS and other public health contexts, whilst high-priced patented medicines may be reimbursed by the public health system, so in theory, accessible. However, high medicine prices can lead to ‘opportunity costs’, where funding high-cost medicines means other treatments cannot be funded by the public health system due to finite public health budgets.^42^ Therefore, such IPRs may impact public access to other health-technologies at the national level.

Accordingly, a tension can arise between the altruistic spirit in which many donors contribute biomaterials to biobanks to benefit society more generally, and how IPRs are controlled by rightsholders and can be used by rightsholders in a way which hinders public access to benefits arising. Dove and Joly have argued the:

…“closed world” IPD [Intellectual Property Discourse], characterized by individual rights, property enclosure, and innovation as an a priori social good, is challenging and being challenged by emerging “open world” BD [Biobanking Discourse] that emphasizes communal rights, reciprocity, solidarity, citizenry, as well biobank-related issues such as genetic sequence databases and open data sharing.^43^

The tension that can arise is not merely a theoretical one. Various empirical studies highlight donors’ concerns around the donation of biomaterials where commercial interests, including IPRs, are involved. For instance, Critchley and others,^44^ state that:

A *commercialisation effect*, defined as a significant decrease in trust or support associated with private relative to public examples, has been found to occur in relation to industry ownership and control of biobanks;^45^ the use of patents;^46^ funding of research,^47^ the type of third party accessing tissue or genomic information;^48^ and selling tissue.^49^

Other studies indicate lower levels of trust by the public in relation to for-profit entities conducting research as opposed to public research institutions,^50^ while other studies recommend greater education of the public on the role of pharmaceutical entities in health research and public–private collaborations in the health context.^51^

In the context of IPRs and biobanks, Nicol and others conducted a study using deliberative democracy methods involving small group discussions amongst participants in Tasmania, which included discussions about various aspects of commercialisation in the biobank context, where the authors found:

Overall, participants expressed what we refer to as a ‘natural prejudice’ against involvement of commercial interests in biobanking and use of biobank resources … Other *concerns were expressed about patenting and monopolizing research results stemming from the use of biobank resources, as well as failure to publish such research*.^52^[Emphasis added]

Yet, these participants recognised the likely expectation and/or need for commercial involvement within the biobanking context, as the authors found that:

Despite this, almost all participants seemed to have an expectation that commercial parties would have to be involved with biobanks in one way or another. This point was exemplified by the following comment by SG3:P2 in his small group discussion: ‘I can’t see any alternative to that commercial mechanism and I’m quite content with that … but I don’t really trust open self-regulating kind of capitalist enterprises either’. For SG3:P2, and, *indeed, for most other participants, appropriate checks and balances need to be in place to ensure that commercialization is managed properly so that the public good focus of biobanks is not lost, …*^53^ [Emphasis added]

Nicol and others found that:

… participants soundly rejected the notion that there should be some financial return to donors in exchange for participation. All but one participant also rejected the notion that there should be any other advantage to donors arising from participation… Rather, they strongly supported the idea that improved treatments should be available to all in need, as this was the primary reason to have a biobank.^54^

This empirical study illustrates some of the concerns members of the public can have about commercial interests within the biobank context, including concerns about how patents (and other IPRs) may impact access to technologies produced/generated in the biobanking context, including health-technologies developed. The study also demonstrates that participants felt a key goal underpinning the biobanking endeavours was to ensure treatments developed would be accessible to all who need them, and the need for checks to ensure the public good mission of the biobank is achieved.

Despite such public concerns arising around commercialisation interests, including IPRs in the biobank context, as the next sections demonstrate, the various legal frameworks within Europe, which could apply in the biobank context, have limited—if any— provisions which: (i) mandate that donors are informed in general terms of how patents (and other IPRs) may arise over downstream health-technologies and how these may potentially impact access to health-technologies developed in certain contexts or (ii) limit how such patents can be used, to safeguard downstream access to patented health-technologies developed/contributed to by knowledge generated via biobank samples.

### A. Informed consent and IPRs

Informed consent is a central component of human health research, often seen as a key component to protecting participants’ broader autonomy and dignity interests.^55^ The importance of informed consent within the health context is reflected in its protection in various international laws and ethical guidelines.^56^ Obtaining informed consent entails that donors must be provided with adequate information so that they are equipped to make autonomous decisions, including in relation to donating biomaterials to a biobank.

There is no tailored legally binding instrument in the UK or Europe addressing the donation of biomaterials for biobanking which specifies in detail what information must be disclosed to obtain adequate consent from donors for the donation of biomaterials to a biobank.^57^ Instead, a piecemeal legal framework applies. For example, in the EU context, several EU laws containing informed consent guidance could apply in the biobanking context, including regulations related to clinical trials and general data protection laws.^58^ However, within these and other contexts, there are no legally binding obligations in the EU or Europe more generally, mandating that the informed consent process for donation to a biobank must include a discussion with biobank donors over how IPRs may arise over and can potentially impact access to downstream health-technologies developed.

Within patent law, the need for informed consent (albeit in the context of biotechnological inventions only) is recognised under Recital 26 of the Biotechnology Directive 98/44EC, which is applicable in all EU States. This Directive has been adopted as ‘supplementary interpretation’ for European Patent Convention States, including the UK. Recital 26 states that:

Whereas if an invention *is based on* biological material of human origin or if it uses such material, where a patent application is filed, the person from whose body the material is taken must have had an opportunity of expressing *free and informed consent* thereto, in accordance with national law. [Emphasis added]

Accordingly, in Europe, biotechnological inventions developed using donated biomaterials require free and informed consent from the donor of that biomaterial. Failure to obtain this could lead to the invalidity of the patent.^59^ However, a narrow interpretative approach has applied to this provision to date; it is not generally used in practice to deny patents.^60^ Moreover, the provision does not specify what information needs to be disclosed to meet informed consent standards, which generally rely on the relevant national standards applicable. Arguably, to fulfil this provision under the current framework, one would merely have to show that a donor consented to the taking of the sample from their body and its use in research.

Nonetheless, existing non-binding guidelines and recommendations encourage disclosing certain information to biobank donors, including disclosure of commercial issues. For instance, the OECD Guidelines on Human Biobanks and Genetic Research Databases (HBGRD) (2009) stipulate that operators of such HBGRDs should, as part of their informed consent policies and depending on the nature of their operations, provide information on their ‘policy with respect to benefit sharing’ and ‘[w]here applicable, the expectation that commercial entities may be granted access to the human biological materials, data and information contained within the HGBRD’s database(s)’.^61^ Paragraph 9C of the guidelines state that the HBGRD biobank operators ‘should have a clearly articulated policy and explicitly indicate to participants whether they and/or the HBGRD retain any rights over the human biological materials and/or data and the nature of such rights’.^62^ While paragraph 9D recommends that operators of biobanks have a ‘clearly articulated policy that is communicated to participants relating to the commercialisation of its own resources, research results derived from those resources, and/or commercial products, if any, that may arise from research using its resources’.^63^ These Guidelines recommend that ‘operators of the HBGRD should have a clearly articulated policy in regards to intellectual property rights, which should address the rights, if any, of the HBGRD, researchers and participants’.^64^ Such provisions represent best practices but do not legally bind OECD Member States or biobanks within their territories. Instead, whether biobanks choose to adopt such provisions is at their discretion. Moreover, informing people of the potential for commercial entities to have access to the samples/data provided to the biobank, or that the results/technologies developed may be commercialised does not necessarily explain to people considering donation how access to such technologies may be affected by commercialisation, including in relation to the potential impacts and role of IPRs over downstream technologies developed in this context. Furthermore, regarding disclosure of IPRs, the OECD guidelines merely state that biobanks should have a *clear policy on IPRs*. This does not specify what information about the potential for IPRs over downstream technologies or how these may potentially affect access to technologies must be shared with people before donation. It is not that IPRs can arise, but rather *how* these IPRs, if they arise, may be used by rightsholder(s) in a manner that could impact downstream access to health-technologies, that would likely be most relevant for donors considering donation if their motivation is to contribute to public health benefits.

The *Council of Europe Recommendation CM/Rec (2016) 6 on Research in Biological Materials of Human Origin* emphasises the need for informed consent in human health research.^65^ It states that donors and patients contributing biological materials should ‘be provided with comprehensible information that is as precise as possible’ regarding: ‘the nature of any envisaged research use and the possible choices’ the donors could exercise; the ‘conditions applicable to the storage of materials, including access and possible transfer policies’ and ‘any relevant conditions governing the use of the materials...’.^66^ However, it does not refer to the need to disclose how IPRs may arise over health-technologies developed. One could take an expansive interpretation of this provision and imply from it that biobanks should disclose potential commercialisation plans (in general terms) related to downstream technologies that may be developed using biomaterials—as the provision requires that ‘any relevant conditions governing the use of the materials’ are disclosed. However, arguably, this approach is unlikely because third-party researchers may oppose such an expansive interpretation in negotiating access to samples. Moreover, the recommendations are not legally binding, so a more minimal interpretation is likely.

In short, there are no legally enforceable European obligations requiring donors of biomaterials to biobanks to be notified, as part of the informed consent process, of the general terms around the potential IPRs that may arise over downstream technologies developed or contributed to by biobank samples, and how these IPRs may potentially impact access to health-technologies arising.

### B. Legal framework around public access to IP-protected downstream health-technologies

Alongside this, if we consider the biobank usage phase, there are no binding European laws that mandate rightsholders to provide donors/biobanks control over IPRs arising or which mandate a requirement to ensure public access to downstream IP-protected technologies developed or contributed to using biobank samples.^67^

There are general guidelines/recommendations encouraging benefit sharing: For example, Article 15 of the United Nations Educational Scientific and Cultural Organisation (UNESCO) Universal Declaration on Bioethics and Human Rights (2005) states that ‘[b]enefits resulting from any scientific research and its applications should be shared with society as a whole and within the international community, in particular with developing countries … benefits may take any of the following forms (a) special and sustainable assistance to, and acknowledgement of, the persons and groups that have taken part in the research; (b) access to quality health care; (c) *provision of new diagnostic and therapeutic modalities or products stemming from research*; (d) support for health services; (e) access to scientific and technological knowledge...’ [Emphasis added].^68^ This Declaration offers a platform to embed benefit sharing in national and regional legal instruments. However, it is not legally binding.

Similarly, the OECD Guidelines on Human Biobanks and Genetic Research Databases (2009) refer to benefit sharing. As a best practice, 9.1 of the Guidelines, states that operators of HBGRD should have clearly articulated benefit-sharing policies which: ‘ … should address, inter alia, whether tests or products arising from research using its resources might be shared with the community and/or the general population, and how such sharing will be effected’.^69^ Moreover, as a best practice, 9.2 of the Guidelines state that HBGRD operators should ‘negotiate benefit sharing agreements before a study begins...’.^70^ However, these Guidelines do not refer to how IPRs over such downstream products should be used by rightsholders or to requirements for safeguarding public access to downstream IP-protected technologies. One could arguably interpret the guidelines to imply that if products arising from research should be shared with the community, then if these technologies are protected by IPRs, there should be a mechanism to step in if the rightsholders use IPRs in a restrictive manner, which limits access, in order to facilitate benefit sharing. However, this would require a broad interpretation of the guidelines, which is unlikely, given their non-binding nature.^71^

The Guidelines recommend as best practice that researchers submit annual progress reports to the HBGRD, including a ‘list publications, published patent applications and patents issued arising from research accessing the HBGRD’s resources’.^72^ However, these recommendations do not extend to providing information to *donors* about patent applications, or any other IPRs sought, and do not entail obligations for biobanks/donors to be informed about how such IPRs are being used or their potential impacts on public access to downstream technologies.

Evident again in such benefit-sharing provisions is the bifurcation within the biobanking process, where donors’ contribution in the donation phase and the intentions/desires of donors are framed around public benefits/altruism, yet this is in dissonance with how downstream health-technologies developed in the usage phase may be protected by IPRs and how such IPRs may potentially impede public access to such technologies.^73^

## IV. BIOBANK DONATION, PUBLIC BENEFITS AND INTELLECTUAL PROPERTY RIGHTS **OVER** DOWNSTREAM TECHNOLOGIES: THE BIOETHICAL IMPLICATIONS

Reflecting on the foregoing analysis, this section makes the case that the current system gives rise to a significant potential for bioethical issues, including: (i) implications for donor autonomy due to the lack of binding obligations to disclose as part of the informed consent process to donors the potential for patents (and other IPRs) to arise over downstream technologies developed, and how, in general terms, these IPRs may impact access to such technologies; and (ii) implications for donors’ dignity interests given that IPRs could be used to impede access to health-technologies, despite donors’ altruistic intentions.

### A. Informed consent, disclosure **around potential for** IPRs and biobanking: donors’ autonomy interests

The need to protect ‘autonomy’ in health research is emphasised in a range of legal instruments and guidelines in Europe and internationally.^74^ Although there is no one recognised definition of ‘autonomy’ within the bioethics or health law fields,^75^ the core concept of ‘autonomy’ centres on the ability of moral agents to make informed and self-directed decisions. The importance around the protection for autonomy within modern bioethics is evident in the emphasis on ‘informed consent, patient rights and the value of people making their own decisions about medical care’.^76^ It extends to ‘privacy, voluntariness, self-mastery, choosing freely, choosing one’s own moral position and accepting responsibility for one’s choices’^77^ and includes considerations of ‘self-control and self-determination’.^78^ In simple terms, autonomy is often seen as an expression of personal liberty.^79^ 

When donors seek to donate biomaterials to a biobank, as part of the informed consent process, donors are provided with information that, in theory, should allow them to make autonomous decisions, thereby safeguarding their decisional autonomy. However, because there are no binding legal principles prescribing specific requirements for the content of the information disclosed as part of the consent process in the biobank context,^80^ the nature of the information provided and the consent process can vary depending on several factors, including the relevant national laws, where applicable.^81^

While disclosure around key components within the consent process including confidentialty, risks for participants, voluntariness etc may assuage concerns about coercion and deception, we argue that the lack of obligations to disclose the potential for IPRs and on how these could potentially impact access to health-technologies developed, is problematic, given the empirical evidence suggests that a key factor motivating donors is an altruistic desire to contribute to public benefits. The fact that access for the public (or certain cohorts of the public) to new health-technologies developed could be hindered by how IPRs may be used is, in our view, likely a ‘material’ factor to such donors’ decisions to donate to the biobank and thus, should be disclosed to donors as part of the informed consent process.

The reasoning in the UK Supreme Court decision in *Montgomery v Lanarkshire^82^* is instructive in this context. This case considered the requirements to disclose risks pertaining to medical treatment in the negligence context.^83^ The *Montgomery case* provided recognition for the need to protect informed consent as part of patients’ broader human right to self-determination and autonomy as enshrined in Article 8, European Convention on Human Rights (ECHR),^84^ and by virtue of the UK’s adoption of the Human Rights Act 1998. To achieve adequate disclosure for medical treatment as required by Article 8 ECHR, the court stated that patients must be made aware of the ‘material risks’ of a treatment and its alternatives. It stated:

The doctor is therefore under a duty to take reasonable care to ensure that the patient is aware of *any material risks* involved in any recommended treatment … *The test of materiality is whether, in the circumstances of the particular case, a reasonable person in the patient’s position would be likely to attach significance to the risk, or the doctor is or should reasonably be aware that the particular patient would be likely to attach significance to it* (Lords Kerr and Reed [2015], UKSC 11, para 87). [Emphasis added]

While the *Montgomery* case did not concern health research, the right to autonomy in health research is also enshrined within human rights instruments, including Article 8 ECHR.^85^ Thus, to vindicate health research participants’ autonomy—at least in the UK under the Montgomery line of reasoning—arguably, health research participants should also be made aware of any material risks where they are participating in health-research that involves interventions on their bodies. Extending this reasoning further, in the biobanking research context, we argue that ‘material factors’ which may impact individual participants’ decisions to donate should—as far as possible—also be disclosed to vindicate such participants’ rights to autonomy.

Given the centrality of donors’ desire to contribute to health benefits in the publicly funded biobank context,^86^ we argue that factors which may impact how such benefits, including technologies developed, are used/accessed by the public, including the potential for future IPRs, would be material factors to donors. Thus, donors should be informed of such factors as part of the informed consent process. We acknowledge that it will not be possible to provide specific information on all potential new technologies or future downstream IPRs that may arise from research on biobank samples, as it is often impossible to predict how research may develop into new technologies. Instead, donors should be made aware in general terms about the potential for IPRs to arise over any downstream technologies, who will hold such IPRs and how such IPRs could *potentially* impact access to such technologies downstream.^87^

A counterargument that could be raised here is that since the likelihood of developing a health-technology from one donor’s biomaterials is limited, it is unnecessary to disclose such information. However, in our view, it is immaterial whether a technology will arise or not based on an individual donor’s contribution. Instead, we must consider the spirit in which donations are made by donors to publicly funded biobanks, and where biobanks and donors’ motivations focus on the potential that the samples may contribute to the development of benefits for the public, including by contributing to the development of new technologies, then how such technologies, if developed, may be controlled—if IPRs arise—is a material factor which should be communicated to donors. Just as one must disclose material risks that may arise before a patient undergoes a medical intervention to satisfy informed consent requirements, we argue that similarly, material factors which may affect how benefits developed using donors’ contributions can be used/accessed downstream should be disclosed in general terms. The fact that we do not know whether a patient will incur a risk when we provide information on a medical procedure to them does not mean we should not disclose such potential risks. Similarly, the fact we cannot know if biomaterials from a particular donor will contribute to a new treatment, or if IPRs will apply or how such IPRs may affect how such technologies are accessed, does not negate the need to disclose in general terms the *potential* IPRs that may arise over treatments developed *and* the potential for these to be used by rightsholders in a manner that impacts the public’s access to such technologies. Many donors may still decide to donate their samples having such information. However, in such cases, they would be doing so with the knowledge that new health-technologies may arise but may be protected by IPRs, which may (in some cases) impact access to such technologies by the public (or some sections of the public) for a period of time. Such donors would thus, be making a more informed choice around donation.

Existing cases demonstrate that informing people about how downstream research is commercialised can impact people’s decisions on whether to donate biomaterials. For example, the *Greenberg v Miami Children’s Hospital*^88^ case concerned the families of children who suffered from Canavan disease and had donated biomaterials and data to aid the development of tests/therapeutics and understandings of the disease. Patents were sought and granted over the isolated gene related to the condition—whose isolation (and the discovery of relevant characteristics of this gene) were contributed to by health research that involved such participants’ biomaterials and data. The patents were allegedly used under exclusive licensing deals to charge high royalty fees, impacting access to testing.^89^ The plaintiffs claimed their understanding was that testing developed:

in connection with the research for which they were providing essential support would be provided on an affordable and accessible basis, and that Matalon’s research would remain in the public domain to promote the discovery of more effective prevention techniques and treatments and, eventually, to effectuate a cure for Canavan disease.^90^

They claimed they were not informed of the intention to patent/commercialise the research,^91^ and had they been made aware of this, they would not have provided the biomaterials on such terms.^92^

In short, not providing sufficient information about IPRs that may arise over downstream technologies developed and how such IPRs may affect access to such technologies means donors have incomplete information as part of the informed consent process, which can have potentially significant implications for donor autonomy.

### B. Donors, biobanks and (public access to) benefits: donors as a means to an end?

The lack of obligations around downstream IPRs can potentially affect donors’ dignity interests by leaving some donors to feel they were used as a ‘means to an end’. ‘Dignity’ is an ambiguous concept with many different conceptualisations.^93^ Here, we focus specifically on Kant’s second maxim of the categorical imperative, which states that: ‘So act that you use humanity, whether in your own person or in the person of any other, always at the same time as an end, never merely as a means’.^94^

Under this maxim, to respect a person’s dignity, that person must not be treated as *merely* a means to an end^95^ because rational (autonomous) persons have inherent value and, as such, are ends in themselves.^96^ Kleingeld notes that the rule is ‘widely understood to mean that there is an absolute moral limit to what we may do to one another (and to ourselves) in the service of our ends, no matter how desirable or important those ends may be’.^97^ To understand whether someone is being treated as a means to an end, the following statement by Kant is often cited:

He who has it in mind to make a false promise to others sees at once that he wants to make use of another human being merely as a means, without the other at the same time containing in himself the end. For, he whom I want to use for my purposes by such a promise cannot possibly agree to my way of behaving toward him, and so contain himself the end of this action.^98^

Van der Graaf and van Delden have interpreted this as indicating that:^99^

agents are used merely as a means when there are no sufficient reasons for them to consent to the action of the person who uses them and they cannot share the end that this other person is pursuing.

Reflecting on this interpretation, the first condition for someone being treated as a means to an end is that ‘ … there are no sufficient reasons for them to consent to the action of the person who uses them’.^100^ This is linked to the notion of a false promise having been made, including where there is information asymmetry between participants involved in the interaction. As Kleingeld states, to be able to give genuine consent, the person granting consent needs to, *inter alia:* ‘have and understand the relevant information. The person needs to know which end you want him to serve, how you plan to use him, what this will require of him and so on’.^101^

Applying this to the biobank context, donors may donate samples seeking to contribute to public benefits/interests. The fact there is no obligation to inform them of how IPRs may impact access to any benefits if developed arguably conceals relevant information about how their contributions will be used. This could be seen as a false promise—because IPRs can impact whether downstream technologies are accessible to the public. This is one component of why some donors could be viewed as being treated as a means to an end.

The second condition is the end-sharing condition, which suggests that:

the subject who is treated in a certain way by an agent must also share the end that the agent is pursuing in order not to be treated merely as a means.^102^

For a person to not be treated as a means to an end, it must be possible for that person—biobank donors in this case—to share in the end goal. However, this goal may not be realised because if technologies are developed and protected by IPRs, depending on how such IPRs are used, such technologies may not be accessible to the public, including donors.

A counterargument here is that IPRs are temporary rights and, in the case of patents, expire after 20 years. Thus, one might argue that even if access to a patented technology is affected by patents initially, the benefits will eventually accrue to the donors/public. However, 20 years is a substantial time in anyone’s life, especially if the technology under IP protection is a lifesaving or preventive treatment. Donors or their families may not survive until the expiration of relevant patents. Moreover, strategies to extend IP protection, including the evergreening of patents and strategic layering of IPRs, can be used.^103^ Hence, failing to disclose that IPRs may arise and how these may affect access, and failing to make it legally binding to have contractual clauses which can be used by biobanks (or other parties) to intervene if IPRs are used in a way that unreasonably limits public access to technologies means donors may not share in the end goal of the endeavour.

Where there is a failure to disclose IPRs which may arise and how these may impact downstream access, and a failure to embed measures to ensure donors/public can access downstream technologies, this could provoke in donors a feeling of being ‘used’ as a means to an end or exploited as a ‘raw material’. Indeed, in *John Moore’s* case,^104^ where Moore’s cells were removed and used for research purposes, and subsequently, a cell line was developed from these, then patented and commercialised without Moore’s knowledge/consent, it was said that:

… he felt that his integrity had been violated, his body exploited, and his tissue turned into a product. He said that his doctors had “claim[ed] that my humanity, my genetic essence, was their invention and their property. They viewed me as a mine from which to extract biological material. I was harvested”.^105^

The failure to disclose the commercialisation plans appeared to evoke in him feelings of being exploited and used.^106^

We acknowledge that in John Moore’s case, there was no knowledge or consent for the research conducted, and his samples contributed to the development of cell lines arising directly from his cells, which were patented and commercialised. These aspects differ from the biobank context, where there will often be a general consent for research using various donors’ biomaterials, but the consent process may have not provided information on the potential for downstream IPRs and how such IPRs may affect access to downstream technologies developed. Moreover, typically in the biobank context, a range of donors’ samples are used by researchers and can contribute to developing useful knowledge and health-technologies. Nonetheless, biobank donors who donate with the specific intention of assisting the public interest and who are not informed that technologies developed may be protected by IPRs that can potentially impede access to technologies may also feel exploited. Effectively, the basis and promise on which they are donating could be seen as not fulfilled, and instead is potentially impeded by the operation of IPRs, in such cases. Hence, we argue that the lack of legally binding obligations to mandate biobanks to disclose potential downstream IPRs that may arise and in general terms explaining how these IPRs may impact access to technologies developed, coupled with the lack of obligations to safeguard reasonable public access to downstream technologies developed, is inadequate to protect donors’ dignity interests.

## V. DOWNSTREAM INTELLECTUAL PROPERTY RIGHTS AND BIOBANK DONATION: AVENUES FOR AMELIORATING THE POTENTIAL BIOETHICAL ISSUES ARISING

To ameliorate the current bioethical issues arising from how IPRs are dealt with for publicly funded biobanks, we need a more holistic approach that embeds greater consideration of donors’ interests from the donation stage to the usage stage. This section puts forward three main proposals for how we could start to achieve this.

### A. Informed consent, donors and biobank participation: mandating requirements for the disclosure around the potential for IPRs

First, it is essential that, as part of the informed consent process, potential donors are informed of how any health-technologies developed may be protected by IPRs, *and* how such IPRs may potentially impact access to such health-technologies. Failure to make donors aware that access to downstream technologies (if protected by IPRs) is impacted by rightsholders’ decisions on how to license such technologies, in our view, could render the disclosure inadequate, particularly for publicly funded biobanks where donors are encouraged to donate based on public benefits which can arise. Due to many donors’ altruistic motivations and desire to contribute towards public benefits, it is likely that how access to such benefits is determined will be a *material factor* for such donors’ decision to donate.

A legally binding requirement should mandate that biobanks include as part of the informed consent process information about what IPRs are, how IPRs may arise over future health-technologies, and how IPRs could affect access to health-technologies that are developed. Making donors aware of this before donation is critical in safeguarding their autonomy in the donation process.^107^ Having such mandated information for biobanks to provide as part of the informed consent process would also address the first element of Kant’s categorical imperative discussed above—as if donors are not informed of the potential for IPRs, and how IPRs may impact access to health-technologies that could engender feelings of being exploited.

Furthermore, it would be a key step in linking the biobank donation phase with the usage phase by informing donors how researchers will use their samples and in general terms of how benefits deriving will be controlled/accessed downstream. Ideally, this requirement would be adopted at a regional European or international level to include more biobanks and increase the likelihood of it being embedded as a norm within the broader international community for biobanking.

### B. Biobanks, IPRs, and public access: normalising biobanks legal avenues to intervene

Secondly, in the usage phase, clear legal steps are needed to safeguard reasonable public access to health-technologies and knowledge developed via the use of biobank samples to address donors' broader dignity interests. The bifurcation of the donation and usage phases hampers this as a donor’s agreement is with the biobank alone, while downstream researchers’ agreements are with the biobank. Thus, there is no legal connection between the donors and researchers using the samples. There is a disconnect between how we discuss public benefits that may arise from the research with donors before donation and how access to such benefits (if developed) are controlled in the usage phase.

This disconnect would not be addressed by individual donors having an individual right to benefit from technologies/profits arising or by having a general right to seek to enforce obligations against third-party researchers using biobank samples. This is primarily because if the intention is to contribute to public benefits, the key aim should be to ensure *public access* to benefits arising. Providing individual rights to benefits to specific donors would not achieve this broader collective aim.^108^ Instead, the collective interests of the public and donors as a collective group need to be protected. A key entity that could achieve this is the biobank, which will often encourage donations based on the public benefits that can arise. Given such promises, they must take active steps to ensure benefits developed are publicly accessible even where such benefits are subject to IPRs.

Here, we are not advocating for biobanks to retain IPRs in downstream technologies developed using biomaterials as this would not ensure accessibility of technologies developed. If researchers (or their employers, depending on agreements in place) could not hold IPRs, this could also limit incentives for the use of biobank samples which could have significant adverse effects.^109^ Instead, legally binding requirements are needed mandating biobanks to impose contractual obligations or access licensing clauses with researchers who use biobank samples that address how downstream IPRs can be used and to ensure reasonable public access to such technologies.^110^ The form such clauses take must balance the need to safeguard access to such benefits for the public with a recognition that under the current health innovation structure IPRs play a role in incentivising certain types of research and translational work.^111^

Biobanks could be mandated to include a licensing clause in IPR agreements and material transfer agreements (MTAs) with researchers, allowing the biobank or other third parties to intervene in cases where downstream health-technologies are rendered inaccessible to the public or certain sections of the public. There are examples of such clauses being used in biobank IP policies.^112^ However, such clauses are at the discretion of biobanks to adopt currently. Furthermore, depending on how such IP licensing clauses are drafted, they can impose high thresholds, meaning they are only invoked in exceptional circumstances.

Jordan, Liddicoat and Liddell’s recent empirical study reviews IP policies of a cohort of large human biobanks, highlighting that five main types of IP licensing practices can be used by biobanks in relation to downstream IPRs developed using biobank samples, namely: ‘(i) non-obstruction clauses; (ii) march-in clauses; (iii) grant-back clauses; (iv) return-of-results clauses; and (v) reach-through clauses’.^113^ They found there was a low uptake of all five types of clauses within biobank IP policies and no uniform norms within the field.^114^ Jordan and others encourage the use of march-in clauses for biobanks, which, in their view, can be useful, including to prevent unreasonable licensing practices. The authors define such clauses as follows:

March-in clauses stipulate that certain user-owned IP must be given to the biobank after a triggering event. What constitutes a triggering event is determined by the biobank, and the triggers typically reflect types of user behavior the biobank wants to avoid.^115^

We argue that a model clause should be designed which would outline the general terms of such a march-in-clause which biobanks should be mandated to adopt. Such a clause would allow biobanks to intervene or ‘march in’, if needed, in a more routine manner, where a triggering event arises, and intervention is necessary in the public interest to facilitate reasonable access to downstream health-technologies developed. This would envisage biobanks being empowered to act in a way that aligns with donors’ participation in the biobanking endeavour: viewing the biobank as a collective resource to generate collective and accessible downstream benefits. Model contractual clauses for MTAs/IP policies could be used to address this for all biobanks. In terms of what the clause should entail—it could indicate that IPRs will be held by researchers/their employers, as per applicable IP rules and contractual provisions in the relevant jurisdiction. However, in our view, it should also provide that biobanks retain: a march-in-clause that would allow the biobank a right to a license of such IPRs on a worldwide, royalty-free basis where the rightsholder uses IPRs in an unreasonably restrictive manner, and the license is necessary in the public interest to facilitate adequate access to health-technologies developed via use of the biobank for the public. A non-exhaustive list of circumstances which could trigger the use of the clause should be given,^116^ and these could include where IPRs are being licensed in a manner that results in the IP-protected technology being priced too high for the public to reasonably access; where rightsholder(s) is refusing to license IPRs in a reasonable manner to third parties etc. Biobanks may need some discretion to add specific additional examples to such clauses which are tailored to the context of each biobank.

Such clauses would exist alongside existing State compulsory licensing provisions which allow States to compulsorily license patents in certain circumstances. However, compulsory licensing should not be viewed as a replacement for the need for march-in-clauses for biobanks for three main reasons: (i) compulsory licenses can only be granted over patents and must be granted on a case-by-case basis patent-by-patent basis^117^; (ii) not all States have fully functioning compulsory licensing systems, and in some instances, these mechanisms can be highly bureaucratic to use,^118^ a march-in-clause could offer an avenue to use IPRs in such cases; (iii) biobanks often encourage donors to donate on the basis of the public interests they can contribute to. Hence, our argument is that biobanks have a stewardship duty to take steps to secure public benefits for donors, which such march-in-clauses could fall within.

We acknowledge that there are concerns around whether IP licensing clauses in favour of biobanks should be sub-licensable, including around the extent to which this could lead to a dilution of the value of IPRs if these were licensed broadly.^119^ We argue, however, that it is important that the biobank can sub-license the IPRs in the health-context, as the unreasonable licensing practice at issue may be around rightsholders refusing to offer reasonable licensing terms for third parties to develop/make a needed medical product. Allowing biobanks to sub-license the IP in certain circumstances will enable them to allow others to produce it in such instances, which biobanks themselves may be unable (and not designed) to do. It also reinforces their role as stewards and facilitators of the public interest in such contexts. However, consideration will be needed by policymakers around how broad such a sub-licensing right should be, and issues including, the extent to which any royalties should be paid by the sub-licensing entity to the rightsholder.^120^

Finally, another concern could be the extent to which biobanks have the ability to monitor how downstream IPRs are used. To address this, we recommend that an additional clause should be provided, which would give third parties (including members of the public) the right to petition the biobank where access to a health-technology developed using samples created by the biobank was not being adequately provided, including where it was unaffordable to the public. This would act as a means for third parties, including donors, their families, the public, civil society groups etc., to petition a biobank in such cases. On foot of such a petition, the biobank would then be under a duty to examine how such IPRs are being used, and to consider whether a march-in-clause should be activated in the circumstances. A clause like this within an IP policy also provides biobanks and the public with an important tool to use as leverage which could be invoked if better terms for access to patented health-technologies are not provided. In this way, it could act as a valuable negotiating tool.

We acknowledge that before such a change can be adopted at the national/international level, certain aspects would need to be considered and developed. Ideally, this would be done following stakeholder consultation. For example, it may be difficult to determine how far a clause of this nature could stretch and how one would define research developed using biobank samples covered by this clause and research which fell outside that. However, this issue is not insurmountable. It could be defined within the policy adopted.

### C. Public and researchers’ awareness of the role of IPRs in health innovation

Alongside such measures, it is vital that we increase public understanding of what IPRs are and their role in health innovation. There is a dearth of empirical evidence about how the public understands IPRs, including the level of public understanding around how such rights can impact health innovation or access to health-technologies for the public. Available evidence suggests such public knowledge is limited. For example, in 2022, the US Intellectual Property Alliance published a study with over 1000 respondents in the USA who were asked about their knowledge of IPRs. The findings suggested respondents had some awareness of IPRs’ role. Still, many could not articulate or define what IP terms mean, respondents also had more limited awareness of issues confronting IP in the USA, and tended to view IPRs positively.^121^ There was no reference to the potential impacts of IPRs on access to health in the survey.^122^ Having greater public awareness about what IPRs are, how the use of IPRs can potentially impact the development and access to health-technologies, and how governments and others can use IPR licensing clauses to shape how IPRs are used could allow greater public input into and pushback around the use of IPRs in a manner that may hinder public access to technologies. This could be incorporated at the national level as part of a national health education campaign. Increasing such public awareness would also increase potential future donors’ awareness of such issues when they consider donating to biobanks.

Furthermore, there is limited research on scientific researchers’ awareness of how IPRs can impact the access to downstream health-technologies. Existing—albeit again limited—studies are available within the general health innovation context, these indicate clinicians/researchers often have a limited understanding of the role of IPRs.^123^ Greater empirical research is needed to investigate the level of understanding scientists have over how IPRs can be used and their impact on access and use of IP protected technologies. Training programmes to increase this knowledge are vital so that scientists/clinicians or relevant research institutions, biobanks,^124^ etc. have a greater awareness of the significance of IPRs in such contexts. Such educational initiatives should be adopted as part of training for biobank operators and scientific researchers using biobanks on how IPRs can be used over technologies developed, how this can impact access to such technologies by the public, and how IP licensing policies/clauses can be used to ameliorate this.

## VI. CONCLUSION

Biobanks’ samples contribute vital knowledge to our understanding of human health and disease and towards developing new health-technologies. Publicly funded biobanks often encourage the public to donate samples under a promise that such samples will contribute to public benefits. Empirical evidence also shows that the desire to contribute to public knowledge and new treatments for the public is a key factor motivating many donors who provide samples to biobanks. Yet, as discussed, when health-technologies are developed—even where samples from biobanks have contributed to such developments—they will often be protected by IPRs, including patents. Such IPRs are held by relevant researchers or in many cases by their employers, not by donors or biobanks, and may be used in a manner which impedes the public’s access to such technologies. In this regard, we see knowledge derived from publicly sourced samples transformed into (or contributing towards) privately held and controlled outputs, including new health-technologies, and due to how IPRs can apply and operate over such technologies, they may be inaccessible for various publics. There is a mismatch between the promise of public benefits in the donation phase and the individualised way IPRs operate over downstream technologies developed.

This article has demonstrated that the current framework gives rise to significant bioethical concerns: The lack of legal obligations to disclose to donors—as part of the informed consent process prior to donation—how IPRs can arise over health-technologies developed *and* how these rights can potentially impact public access to health-technologies can impact donors autonomy. Moreover, this lack of information, combined with the lack of legal obligations mandating clauses to allow interventions with such IPRs if access is unreasonably restricted, can leave donors feeling like a means to an end, impacting their dignity. Accordingly, an urgent reconsideration is needed around the (lack of) legal framework(s) around governing how we communicate—with donors and the public—about the potential for IPRs *and* specifically, with how these may impact access to downstream health-technologies developed in the biobank context; and around the mechanisms to intervene with rightsholder(s) control where public access to downstream benefits is unreasonably hindered in such contexts.

## Footnotes

1 See generally: Sameer Al Diffalha and others, ‘The Importance of Human Tissue Bioresources in Advancing Biomedical Research’ (2019) 17 Biopreservation and Biobanking 209–12; Marianna J Bledsoe and William E Grizzle, ‘The Use of Human Tissues for Research: What Investigators Need to Know’ (2022) 50 Alternative to Laboratory Animals 265–74.

2 Organisation for Economic Co-operation and Development (OECD), ‘Glossary of Statistical Terms’ (OECD 2007) 68. <https://ec.europa.eu/eurostat/ramon/coded_files/OECD_glossary_stat_terms.pdf> accessed 20 March 2023.

3 European Commission, ‘Biobanks for Europe: A Challenge for Governance’ (European Commission 2012) 15. <https://www.coe.int/t/dg3/healthbioethic/Activities/10_Biobanks/biobanks_for_Europe.pdf> accessed 20 March 2023

4 ibid 14.

5 On the potential benefits of biobanks for the public interest, see discussion in: Graeme Laurie and others, ‘Managing Access to Biobanks: How Can We Reconcile Individual Privacy and Public Interests in Genetic Research?’ (2010) 10 Medical Law International 315–37, 322; See also: Shawn HE Harmon and Aisling M McMahon, ‘Banking (On) the Brain: From Consent to Authorisation and the Transformative Potential of Solidarity (2014) 22 Medical Law Review 572–606, 581.

6 In using the terms ‘public’ and ‘public interest’ we recognise that there are different groups within society, with different needs and interests, and we, therefore, use these terms as shorthand in this article to encompass various different publics, and differing public interests within society.

7 For a discussion of IPRs in the biobank context, see: Saminda Pathmasiri and others, ‘Intellectual Property Rights in Publicly Funded Biobanks: Much Ado About Nothing?’ (2011) 29 Nature Biotechnology 319; Naomi Hawkins, ‘Intellectual Property and Biobanks’ in Deborah Mascalzoni (ed), *Ethics, Law and Governance of Biobanks* (Springer 2015) 39–52; Aisling McMahon, ‘Patents, Human Biobanks and Access to Health Benefits: Bridging the Public–Private Divide’ in Jessica Lai and Antoinette Maget Dominicé (eds), *Intellectual Property and Access to Im/Material Goods* (Edward Elgar 2016); Kathleen Liddell, John Liddicoat and Matthew Jordan, ‘IP Policies For Large Bioresources: The Fiction, Fantasy, and Future of Openness’ in Timo Minssen, Janne Rothmar Herrmann and Jens Schovsbo (eds), *Global Genes, Local Concerns: Legal, Ethical, and Scientific Challenges in International Biobanking* (Edward Elgar 2019) 242–63; Timothy Caulfield and others, ‘Evidence and Anecdotes: An Analysis of Human Gene Patenting Controversies’ (2006) 24 Nature Biotechnology 1091–94; Timothy Caulfield and Blake Murdoch, ‘Genes, Cells, and Biobanks: Yes, there’s still a Consent Problem’ (2017) 15 PLOS Biology 1–9; Rajam Neethu, ‘Biobank-related Research and Intellectual Property Rights: Deconstructing the Obscurity’ (2017) 12 Journal of Intellectual Property Law & Practice 307–20; Hermann Garden, Naomi Hawkins and David Winickoff, ‘Building and Sustaining Collaborative Platforms in Genomics and Biobanks for Health Innovation’ (2021), OECD Science, Technology and Industry Policy Papers, No 102, 37–38.

8 See discussion in: Hawkins (n 7), at 44–45.

9 Although there are limited exceptions, donors generally have no rights in separated biomaterials. See Muireann Quigley, ‘Property in Human Biomaterials—Separating Persons and Things?’ (2012) 32 Oxford Journal of Legal Studies 659–83. On the limited control donors have in this context, see Roger Brownsword, ‘Biobank Governance: Property, Privacy And Consent’ in Christian Lenk, Nils Hoppe and Roberto Andorno (eds), *Ethics and Law of Intellectual Property: Current Problems in Politics, Science and Technology* (Ashgate 2006); Donna Dickenson*, Property in the Body: Feminist Perspectives* (CUP 2017) 115–22.

10 For a discussion of these potential tensions, see: Edward S Dove and Yann Joly, ‘The Contested Futures of Biobanks and Intellectual Property’ (2012) 11 Theory & Law: Journal of Legal Thought 132; Nadja Kanellopoulou, ‘Reciprocity, Trust and Public Interest in Research Biobanking: In Search of a Balance’ in Christian Lenk and others (eds), *Human Tissue Research: A European Perspective on the Ethical and Legal Challenges* (OUP 2011) 45–53; McMahon (n 7).

11 Pathmasiri and others (n 7); Hawkins (n 7); McMahon (n 7).

12 McMahon (n 7); See also discussions in: Hawkins (n 7); Pathmasiri and others (n 7).

13 For a discussion of such issues, see Ellen ‘t Hoen, ‘Private Patents and Public Health: Changing Intellectual Property Rules for Access to Medicines’ (Health Action International, 2016); Karen Walsh and others, ‘Intellectual Property Rights and Access in Crisis’ (2021) 52 IIC 379–416; Thomas Pogge, Matthew Rimmer and Kim Rubenstein (eds), *Incentives for Global Public Health: Patent Law and Access to Essential Medicines* (CUP 2010).

14 See overview in: Jan Domaradzki and Jakub Pawlikowski, ‘Public Attitudes toward Biobanking of Human Biological Material for Research Purposes: A Literature Review’ (2019) 16 International Journal Environmental Research Public Health 2209 <https://doi.org/10.3390/ijerph16122209> accessed 7 July 2023; who cite the following: Åsa Kettis-Lindblad and others, ‘Genetic Research and Donation of Tissue Samples to Biobanks. What do Potential Sample Donors in the Swedish General Public Think?’ (2006) 16 European Journal Public Health 433–40; Casey Lynette Overby and others, ‘Prioritizing Approaches to Engage Community Members and Build Trust in Biobanks: A Survey of Attitudes and Opinions of Adults within Outpatient Practices at the University of Maryland’ (2015) 5 J Personalized Med 264–79; Manjulika Vaz, Mario Vaz and K Srinivasan, ‘Listening to the Voices of the General Public in India on Biomedical Research—An Exploratory Study’ (2015) 12 Indian Journal Medicine Ethics 68–77; Mary Dixon-Woods and others, ‘A Qualitative Study of Participants’ Views on Re-consent in a Longitudinal Biobank’ (2017) 18 BMC Medical Ethics 22.

15 Domaradzki and Pawlikowski, ibid, 3.4.

16 As cited in ibid: K Hoeyer, ‘Donors Perceptions of Consent to and Feedback from Biobank Research: Time to Acknowledge Diversity?’ (2010) 13 Public Health Genomics 345–52; AA Lemke and others, ‘Public and Biobank Participant Attitudes toward Genetic Research Participation and Data Sharing’ (2010) 13 Public Health Genomics 368–77; Yi Ma and others, ‘Consent for Use of Clinical Leftover Biosample: A Survey among Chinese Patients and the General Public’ (2012) 7 PLoS One 1–7; Pamela Tozzo, Anotnio Fassina and Luciana Caenazzo, ‘Young People’s Awareness on Biobanking And DNA Profiling: Results of a Questionnaire Administered to Italian University Students’ (2017) 13 Life Science Society and Policy 1–12; Hélène Nobile and others, ‘Why do Participants Enrol in Population Biobank Studies? A Systematic Literature Review’ (2013) 13 Expert Review of Molecular Diagnostics 35–47.

17 As cited in Domaradzki and Pawlikowski (n 14); Lemke and others, ibid; Maile Tauali’I and others, ‘Native Hawaiian Views on Biobanking’ (2014) 29 Journal of Cancer Education 570–76; Nobile and others, ibid.

18 As cited in Domaradzki and Pawlikowski (n 14); Evette J Ludman and others, ‘Glad you Asked: Participants’ Opinions of Re-Consent for dbGaP Data Submission’ (2010) 5 Journal of Empirical Research on Human Research Ethics 9–16; Susan S Khalil and others, ‘Attitudes, Understanding, and Concerns Regarding Medical Research Amongst Egyptians: A Qualitative Pilot Study’ (2007) 8 BMC Medical Ethics 1–12; Kathryn L Braun and others, ‘Cancer Patient Perceptions about Biobanking and Preferred Timing of Consent’ (2014) 12 Biopreservation and Biobanking 106–12.

19 As cited in Domaradzki and Pawlikowski (n 14): Christian Simon and others, ‘Active Choice but Not Too Active: Public Perspectives on Biobank Consent Models’ (2011) 13 Genetics in Medicine: Official Journal of the American College of Medical Genetics 821–31; Jill M Pulley and others, ‘Attitudes and Perceptions of Patients Towards Methods of Establishing a DNA Biobank’ (2008) 9 Cell Tissue Bank 55–65; Amy L McGuire and others, ‘DNA Data Sharing: Research Participants’ Perspectives’ 2008 1 Genetics in Medicine 46–53.

20 As cited in Domaradzki and Pawlikowski (n 14): Celine Lewis and others, ‘Public Views on the Donation and use of Human Biological Samples in Biomedical Research: A Mixed Methods Study’ (2013) 3 BMJ Open 1–11; Natalia I Heredia and others, ‘Community Perceptions of Biobanking Participation: A Qualitative Study among Mexican-Americans in Three Texas Cities’ (2017) 20 Public Health Genomics 46–57; Corinna Porteri and others, ‘Public’s Attitudes on Participation in a Biobank for Research: An Italian Survey’ (2014) 15 BMC Medical Ethics 81; Leena Merdad and others, ‘Assessment of Knowledge about Biobanking among Healthcare Students and their Willingness to Donate Biospecimens’ (2017) 18 BMC Medical Ethics 32.

21 As cited in Domaradzki and Pawlikowski (n 14): Alanna Kulchak Rahm and others, ‘Biobanking for Research: A Survey of Patient Population Attitudes and Understanding’ (2013) 4 Journal of Community Genetics 445–50; Maile Tauali’i and others (n 17); Nobile et al (n 16).

22 As cited in Domaradzki and Pawlikowski (n 14): Amy A Lemke, Colin Halverson and Lainie Friedman Ross, ‘Biobank Participation and Returning Research Results: Perspectives from a Deliberative Engagement in South Side Chicago’ (2012) Part A (158A) 5 American Journal of Medical Genetics 1029–37.

23 Kettis-Lindblad and others (n 14) 435.

24 ibid 435–36.

25 See Dove and Joly (n 10); McMahon (n 7).

26 See discussion in: McMahon (n 7).

27 UK Biobank, ‘Homepage’ (ukbiobank.com, 21 March 2023) <https://www.ukbiobank.ac.uk/> accessed 21 March 2023.

28 Parkinson’s UK Brain Bank, ‘Parkinson’s UK Brain Bank donor information booklet’ (2018) <https://www.parkinsons.org.uk/sites/default/files/2020-03/Parkinson%27s%20UK%20Brain%20Bank%20donor%20information%20booklet%202020_0.pdf> accessed 27 March 2023.

29 National Irish COVID Biobank, ‘Get Involved’ (covidbiobank.ie, 21 March 2023) <https://www.covidbiobank.ie/get-involved> accessed 21 March 2023.

30 BBMRI-ERIC, ‘About Us’ (bbmri-eric.eu, 21 March 2023) <https://www.bbmri-eric.eu/about/> 21 March 2023.

31 For a general discussion of IPRs that can arise in the biobank context, see Pathmasiri and others (n 7) 319–23; On the types of IPRs over downstream technologies, see McMahon (n 7); Timo Minssen, Michiel Verlinden and Issabelle Huys, ‘IPRs in Biobanking—Risks and Opportunities for Translational Research’ (2015) 2 Intellectual Property Quarterly 106–29; Rajam Neethu, ‘Biobank-Related Research and Intellectual Property’ (2017) 12 Journal of Intellectual Property Law & Practice 307–20; Hawkins (n 7) 1.

32 See Siva Thambisetty and others, ‘Addressing Vaccine Inequity during the COVID-19 Pandemic: The TRIPS Intellectual Property Waiver Proposal and Beyond’ (2022) 81 Cambridge Law Journal 384–416; See also: Ove Granstrand, *The Economics and Management of Intellectual Property: Towards Intellectual Capitalism* (Edward Elgar 1999) 249.

33 Article 33, Agreement on Trade-Related Aspects of Intellectual Property Rights (TRIPS Agreement) (15 April 1994) 1869 UNTS 299, Annex 1C.

34 For example, see: Article 53(a), European Patent Convention 1973, as amended, which excludes inventions from patentability where their commercial exploitation is against *ordre public*/morality.

35 Many employment contracts specify that any IPRs created in the course of employment are held by the employer.

36 McMahon (n 7). The human body itself is not patentable (art 5, Biotechnology Directive), but patents are available in Europe on ‘isolated elements of the body’ including isolated human genes. It is the inventor who isolates the gene from the body, and not the person whose body it was taken from who holds the patents. See discussion in: Aisling McMahon, ‘Gene Patents and the Marginalisation of Ethical Issues’ (2019) 41 European Intellectual Property Review 608–20.

37 See discussion in Brownsword (n 9).

38 See discussion in McMahon (n 7).

39 See discussion in: Aisling McMahon, ‘Global Equitable Access to Vaccines, Treatments and Diagnostics for Covid-19: The Role of Patents as Private Governance’ (2021) 47(3) Journal of Medical Ethics 142–48; See also discussion in context of human genome editing technologies in: Duncan Matthews and others, ‘The Role of Patents and Licensing in the Governance of Human Genome Editing: A White Paper’ (30 July 2021). Queen Mary Law Research Paper No 364/2021, Available at SSRN: <https://ssrn.com/abstract=3896308> accessed 7 July 2023.

40 Robin Feldman, ‘May your Drug Price be Evergreen’ (2018) 5 Journal of Law and Biosciences 590–647; Ove Granstrand and Frank Tietze, ‘IP Strategies and Policies for and against Evergreening’ (2014) CIM Working Paper 4 1–37; Reed F Beall and others, ‘Is Patent “Evergreening” Restricting Access to Medicine/Device Combination Products?’ (2016) 11(2) PLOS ONE e0148939.

41 For recent discussions, see John Gapper, ‘The Painfully High Price of Humira is Patently Wrong’ (ft.com 24 February 2023) <https://www.ft.com/content/a8685c8d-60da-4fe8-9bba-ec1ba3bf62cc> accessed 21 March 2023.

42 See discussion in: Donato Paolo Mancini, ‘AbbVie Accused of Overcharging Dutch for Flagship Drug Humira’ (ft.com 21 February 2023) <https://www.ft.com/content/58707816-6332-450e-bcbf-673203176cc0> accessed 21 March 2023.

43 Edward S Dove and Yann Joly, ‘The Contested Futures of Biobanks and Intellectual Property’ (2012) 11 Theory & Law: Journal of Legal Thought 132, 143.

44 Christine R. Critchley and others, ‘Identifying the nature and extent of public and donor concern about the commercialisation of biobanks for genomic research’ (2021) 29 European Journal of Human Genetics 503–511, 505.

45 Christine R Critchley, Dianne Nicol and Margaret Otlowski, ‘The Impact of Commercialisation and Genetic Data Sharing Arrangements on Public Trust and the Intention to Participate in Biobank Research’ (2015) 18 Public Health Genomics 160–72; Dianne Nicol and others, ‘Understanding Public Reactions to Commercialization of Biobanks and use of Biobank Resources’ (2016) 162 Social Science Medicine 79–87, as cited in Critchley and others, ibid, 503–511, 505.

46 EF Einsiedel, ‘Public Perceptions of Transgenic Animals’ (2005) 24 Rev Scientifique et Tech-Off Int Des Epizooties 149, as cited in Critchley and others, ibid, 505.

47 Timothy Caulfield, Christen Rachul and Erin Nelson, ‘Biobanking, Consent, and Control: A Survey of Albertans on Key Research Ethics Issues’ (2012) 10 Biopreser Biobank 433–38; Zubin Master and others, ‘Cancer Patient Perceptions on the Ethical and Legal Issues Related to Biobanking’ (2013) 6 BMC Medical Genomics 8, as cited in Critchley and others, ibid, 505.

48 Critchley, Nicol and Otlowski (n 44); Nicol and others (n 44), as cited in Critchley and others, ibid, 505.

49 K Spector-Bagdady and others, ‘Encouraging Participation and Transparency in Biobank Research’ (2018) 37 Health Aff. 1313–20, as cited in Critchley and others, ibid, 505.

50 Celine Lewis and others, ‘Public Views on the Donation and use of Human Biological Samples in Biomedical Research: A Mixed Methods Study’ (2013) 3 BMJ Open e003056. D, 10, citing: Aaro Tupasela and others, ‘Attitudes towards Biomedical use of Tissue Sample Collections, Consent, and Biobanks among Finns’ (2010) 38 Scandinavian Journal of Public Health 46–52; Gill Haddow and others., ‘Generation Scotland: Consulting Publics and Specialists at an Early Stage in a Genetic Database’s Development’ (2008) 18 Critical Public Health 139–49; Danijela Budimir and others, ‘Ethical Aspects of Human Biobanks: A Systematic Review’ (2011) 52 Croatian Medical Journal 262–79.

51 Celine Lewis and others, ‘Public Views on the Donation and use of Human Biological Samples in Biomedical Research: A Mixed Methods Study’ (2013) 3 BMJ Open e003056. D, 10.

52 Nicol and others (n 45) 83.

53 ibid 83.

54 ibid 83.

55 Rebbeca Roache, ‘Why is Informed Consent Important?’ (2014) 40 Journal of Medical Ethics 435–36; John Coggon and Jose Miola, ‘Autonomy, Liberty and Medical Decision Making’ (2011) 70 Cambridge Law Journal 523–47; For a critique on some of the limits of informed consent, see Onora O’Neil, ‘Some Limits of Informed Consent’ (2003) 28 Journal of Medical Ethics 4–7.

56 In the health research context, these include: The Nuremberg Code (1947); World Medical Association, Declaration of Helsinki: Ethical Principles for Medical Research Involving Human Subjects (1964); The National Commission for the Protection of Human Subjects of Biomedical and Behavioural Research, The Belmont Report: Ethical Principles and Guidelines for the Protection of Human Subjects of Research (1979); World Health Organization, ‘Guidelines for Good Clinical Practice (GCP) for Trials on Pharmaceutical Products’ (1995) WHO Technical Report Series, No 850; Council of Europe, Convention for the Protection of Human Rights and Dignity of the Human Being with regard to the Application of Biology and Medicine: Convention on Human Rights and Biomedicine (1 December 1999) ETS No 164; Council for International Organizations of Medical Sciences (CIOMS) and World Health Organization (WHO), ‘International Ethical Guidelines for Health-related Research Involving Humans’ (2016) (CIOMS Publication No 115).

57 On consent in the biobanking context, see generally: Jane Kaye and others, ‘Consent for Biobanking: The Legal Frameworks of Countries in the BioSHaRE-EU Project’ (2016) 14 Biopreservation and Biobanking 195, 196.

58 For clinical trials, this includes Clinical Trial Regulation (EU Regulation No 536/2014) which entered into force in January 2022 and has provisions around clinical trials and informed consent that may be relevant to biobanks if a biobank is established as part of the trial. This regulation does not apply in the UK. The EU General Data Protection Regulation (GDPR) may also have an impact. The GDPR was adopted as domestic law in the UK via the Data Protection Act 2018 but the UK has discretion to keep this framework under review.

59 Asa Hellstadius and Jens Schovsbo, ‘You Told Me, Right? - Free and Informed Consent in European Patent Law’ in T Minssen, J Rothmar Herrmann and Jens Schovsbo (eds), *Global Genes, Local Concerns: Legal, Ethical and Scientific Challenges in International Biobanking* (Edward Elgar 2019) 92–116.

60 See discussion in: Cliona Kelly and Rachel Claire Brady, ‘Research Ethics and the Patent System’ (2022) 44 European Intellectual Property Review 209–20.

61 Organisation for Economic Co-operation and Development (OECD), ‘Guidelines on Human Biobanks and Genetic Research Databases’ (2009) (OECD Publishing) p 30; See also discussion in: McMahon (n 7).

62 ibid, para 9C.

63 ibid, para 9D.

64 Ibid, para 9E.

65 Council of Europe, ‘Recommendation CM/Rec (2016) 6 of the Committee of Ministers to Member States on Research on Biological Materials of Human Origin’ (2016).

66 art 10(1), ibid.

67 On benefit sharing and biobanking generally, see: Diane Nicol and Christine Critchley, ‘Benefit Sharing and Biobanking in Australia’ (2011) 21 Public Understanding of Science 534–55.

68 UNESCO, Universal Declaration on Bioethics and Human Rights, art 15.

69 OECD Guidelines on Human Biobanks and Genetic Research Databases (2009), 9.1.

70 ibid, 9.2.

71 On the role of benefit sharing as a principle and critique of its effectiveness in context of human health research using biological samples, see Doris Schroeder and Julie Cook Lucas, ‘Towards Best Practice for Benefit Sharing Involving Access to Human Biological Resources: Conclusions and Recommendations’ in Doris Schroeder and Julie Cook Lucas (eds), *Benefit Sharing From Biodiversity to Human Genetics* (Springer 2013) 224.

72 OECD Guidelines on Human Biobanks and Genetic Research Databases (2009), 9.3.

73 Some national EU Member States have adopted legal mechanisms to regulate domestic biobanks, however, there is also often limited engagement with benefit-sharing in the IPR context in such instruments.

74 For example, see: art 6, Universal Declaration on Bioethics and Human Rights (adopted on 19 October 2005, entered into force on 11 March 2006) (UNESCO) UN Doc C/CN.4/2005/CRP.2/Rev.1; art 5, Council of Europe, Convention for the Protection of Human Rights and Dignity of the Human Being with regard to the Application of Biology and Medicine: Convention on Human Rights and Biomedicine (signed 4 April 1997, entered into force 1 December 1999) ETS No 164; The Belmont Report: (n 55); Clinical Trials Regulation (EU) 536/2014 of the European Parliament and of the Council of 16 April 2014 on clinical trials on medicinal products for human use, OJ L 158/1 (2014); World Medical Association, ‘WMA Declaration of Taipei on Ethical Considerations regarding Health Databases and Biobanks’ (2016) (adopted by the 66th WMA General Assembly, Taipei, Taiwan).

75 See discussion in: Louise Campbell, ‘Kant, Autonomy and Bioethics’ (2017) 2 Ethics, Medicine and Public Health 383.

76 David DeGrazia and Joseph Millum, *A Theory of Bioethics* (CUP 2021) 97.

77 Ruth Faden and Tom L Beauchamp, *The History and Theory of Informed Consent* (OUP 1986) 7.

78 Onora O’Neill, *Autonomy and Trust in Bioethics* (CUP 2002) 22.

79 Tom Beauchamp and J Childress, *Principles of Biomedical Ethics* (8th edn, OUP 2019).

80 In the biobank context, there are debates on the type of consent that should be used, these are beyond the scope of this article, see discussions in: Timothy Caulfield and Jane Kaye, ‘Broad Consent in Biobanking: Reflections on Seemingly Insurmountable Dilemmas’ (2009) 10 Medical Law International 85–100; Mark Sheehan, ‘Can Broad Consent be Informed Consent?’ (2011) 4 Public Health Ethics 226–35.

81 Jane Kaye and others, ‘Consent for Biobanking: The Legal Frameworks of Countries in the BioSHaRE-EU Project’ (2016) 14 Biopreservation and Biobanking 195, 196.

82 [2015] UKSC11.

83 The case involved birth injuries to a baby which arose due to shoulder dystocia arising during a vaginal delivery and related to failure to disclose this risk to the patient given her circumstances. See discussion in: TT Arvind and Aisling McMahon, ‘Responsiveness and the Role of Rights in Medical Law: Lessons from Montgomery’ (2020) 28 Medical Law Review 445–77.

84 [2015], UKSC 11, para 87. See discussion in: Jean V Mchale, ‘Innovation, Informed Consent, Health Research and the Supreme Court: Montgomery V Lanarkshire—A Brave New World?’ (2017) 12 Health Economics, Policy and Law 435, 447–48 where McHale states: ‘Montgomery is rooting informed consent in individual human rights. It is recognising the particular characteristics of the individual in relation to information disclosure which is truly rooted in specificity of decision making, which is fundamentally seen as part of human rights and of Article 8 of the ECHR’.

85 See discussion in: McHale, ibid 437; Aurora Plomer, *The Law and Ethics of Medical Research: International Bioethics and Human Rights* (Cavendish Publishing 2005); Other obligations focused on the need for consent and protection of autonomy in the health research include: Declaration of Helsinki (para 26); Council of Europe Convention on Human Rights and Biomedicine (art 5).

86 See nn 14–24.

87 Alternatively, a dynamic consent model could be adopted. For a discussion of dynamic consent and biobanks, see Esther van Zimmeren, ‘Generating Trust in Biobanks within the Context of Commercialization: Can Dynamic Consent overcome Trust Challenges?’ in Timo Minssen, Janne R Herrmann, and Jens Schovsbo (eds), *Global Genes, Local Concerns Legal, Ethical, and Scientific Challenges in International Biobanking* (Elgar 2019) 130–55.

88
*Greenberg v Miami Children’s Hospital Research Institute*, 264 F. Supp. 2d 1064 (S.D. Fla. 2003).

89 See discussion in: McMahon (n 7).

90
*Greenberg v Miami Children’s Hosp* (n 88) 1067.

91 ibid 1068.

92 ibid 1072. Similar issues are discussed, in *Moore v Regents of University of California* 51 Cal.3d 120 (1990).

93 See discussion in: Anna Pacholczyk and Udo Schuklenk, ‘Dignity’s “Wooly Uplift”’ (2010) 24 Bioethics 1–2; Adam Schulman, ‘Bioethics and the Question of Human Dignity’ in Edmund D Pellegrino, Adam Schulman and Thomas W Merrill (eds), *Human Dignity and Bioethics: Essays Commissioned by the President’s Council on Bioethics* (President’s Commission on Bioethics 2008); Ruth Macklin, ‘Dignity is a Useless Concept’ (2003) 327 BMJ 1419; Doris Schroeder, ‘Dignity: Two Riddles and Four Concepts’ (2008) 17 Cambridge Quarterly of Healthcare Ethics 230, 232.

94 Immanuel Kant, *Practical Philosophy* (Mary J Gregor ed tr, CUP 1996) cited in Van der Graaf R and van Delden JJ, ‘On Using People Merely as a Means in Clinical Research’ (2012*)* 26 Bioethics 76, 77.

95 See Louise Campbell, ‘Kant, Autonomy and Bioethics’ (2017) 2 Philosophical Considerations 381, 387; Paulo Henrique Burg Conti and Paulo Vinicius Sporleder de Souza, ‘Bioethics and its Theoretical Paradigms’ (2021) 29 Revista Bioetica 716, 720; Iain Brassington, ‘The Concept of Autonomy and its Role in Kantian Ethics’ (2012) 12 Cambridge Quarterly of Healthcare Ethics 166–76; Onora O’Neill, *Autonomy and Trust in Bioethics* (CUP 2002); David DeGrazia and Joseph Millum, *A Theory of Bioethics* (CUP 2021) 54.

96 Immanuel Kant, *The Moral Law or Kant’s Groundwork of the Metaphysic of Morals* (HJ Patton ed, Hutchinson University Library 1966) 94–96.

97 Pauline Kleingeld, ‘How to Use Someone “Merely as a Means”’ (2020) 25 Kantian Review 389.

98 Immanuel Kant, *Metaphysik der Sitten* (The metaphysics of morals) (1797) In Die Königlich Preußischen Akademie der Wissenschaften ed. 1914. Kant’s gesammelte Schriften. Berlin: Reimer; 6: 462 as cited in Rieke van der Graaf and Johannes JM van Delden, ‘On Using People Merely as a Means in Clinical Research’ (2012*)* 26 Bioethics 76, 78.

99 van der Graaf and van Delden ibid 78; See also discussion in: Samuel Kerstein, ‘Treating Persons as Means’ The Stanford Encyclopedia of Philosophy (Summer 2019 Edition), Edward N Zalta (ed), <https://plato.stanford.edu/archives/sum2019/entries/persons-means/> accessed 7 March 2023; Alexander A Guerrero, ‘Appropriately Using People Merely as a Means’ (2016) 10 Criminal Law, Philosophy 777–94.

100 van der Graaf and van Delden (n 98) 79.

101 Pauline Kleingeld, ‘How to Use Someone “Merely as a Means”’ (2020) 25 Kantian Review 404.

102 van der Graaf R and van Delden JJ (n 98) 78, citing: S. Kerstein, Treating Others Merely as Means’ (2009) 21(2) Utilitas 163–180; C.M. Korsgaard, Creating the kingdom of ends (Cambridge University Press 1996); A.W. Wood, Kant’s ethical thought (Cambridge University Press 1999).

103 See (n 32) and (n 40).

104
*Moore v Regents of University of California*, 793 P.2d 479 (Cal. 1990).

105 Lori Andrews, ‘Who Owns Your Body? A Study in Literature and Law’ (2009) 84 Chicago Kent Law Review 3, 5, citing: John Vidal & John Carvel, Lambs to the Gene Market, Guardian, Nov. 12, 1994, 25.

106 See also discussions in the context of the Henrietta Lacks case: Jenna Greene, ‘Henrietta Lacks’ heirs sue for cut of medical research profits. Are they too late?’ (*Reuters*, 12 April 2022) <https://www.reuters.com/legal/litigation/henrietta-lacks-heirs-sue-cut-medical-research-profits-are-they-too-late-2022-04-12/> accessed 7 July 2023; Craig leMoult, ‘Thermo Fisher seeks Dismissal of Henrietta Lacks’ Family’s Lawsuit Regarding Sale of her Cells’ (*GBH*, 17 May 2022) <https://www.wgbh.org/news/local-news/2022/05/17/thermo-fisher-seeks-dismissal-of-henrietta-lacks-familys-lawsuit-regarding-sale-of-her-cells> accessed 7 July 2023; See also: JoAnne Belisle, ‘Recognizing a Quasi-Property Right in Biomaterials’ (2013) 3 UC Irvine Law Review 767, 771–72.

107 There are existing regulations in the US context, such as the Federal Policy for the Protection of Human Subjects (often called the Common Rule) (as amended) 2018 which mandates disclosure of potential future commercial interests that may arise, including from secondary uses in specific contexts. See Kayte Spector-Bagdady and others, ‘Encouraging Participation and Transparency in Biobank Research’ (2018) 37 Health Affairs 1313–20 <https://www.healthaffairs.org/doi/full/10.1377/hlthaff.2018.0159> accessed February 2024.

108 There are other reasons why individual rights for donors to IPRs/profits may not be desirable or practical, see discussion in McMahon (n 7) 4.3; Hawkins (n 7) 45.See also: Mary Taylor Danforth, ‘Cells, Sales, and Royalties: The Patient’s Right to a Portion of the Profits’ (1988) 6 Yale Law and Policy Review 179–202; Charlotte H Harrison, ‘Neither Moore nor the Market: Alternative Models for Compensating Contributors of Human Tissue (2002) 28 American Journal of Law & Medicine 87.

109 On rationales for not granting IPRs to biobanks in such contexts more generally, see discussion in: McMahon (n 7) 4.2; Pathmasiri and others (n 7) 320.

110 For a general discussion of such clauses, see Aisling McMahon, ‘Accounting for Ethical Considerations in the Licensing of Patented Biotechnologies and Health-Related Technologies: A Justification’ in Naomi Hawkins (eds), *Patenting Biotechnological Innovation: Eligibility, Ethics and Public Interest* (Edward Elgar 2022); in the biobank context, see also discussion in: Hawkins (n 7) 48–29.

111 ibid.

112 See, for example, the UK Biobank Access Procedures (2022), para B12.11 states that: ‘In the event that conduct is considered unreasonably restrictive by UK Biobank, it reserves the right to require that a licence of such rights is granted back to UK Biobank on an irrevocable, perpetual, worldwide, fully paid-up, royalty-free, fully sub-licensable basis so that other researchers who are granted access to use the Resource can exercise such rights to the extent necessary to conduct their research project. *Of note is that this is not a step that UK Biobank has had to invoke over the past 9 years*.’ [Emphasis added] <https://www.ukbiobank.ac.uk/media/llupxihh/20210309-access-procedures-v2-0-final.pdf> accessed 7 July 2023.

113 Matthew Jordan, John Liddicoat and Kathleen Liddell, ‘An Empirical Study of Large, Human Biobanks: Intellectual Property Policies and Financial Conditions for Access’ (2021) 8 Journal of Law and the Biosciences 1–32, 31.

114 ibid 31.

115 ibid, 5.

116 Jordan, Liddicoat and Liddell, also discuss a range of examples that could be provided by biobanks, see ibid 24.

117 McMahon (n 39).

118 Thambisetty and others (n 32).

119 Jordan, Liddicoat and Liddell (n 113) 26 discusses this in the context of grant-back clauses.

120 See discussion, ibid.

121 US Intellectual Property Alliance, Intellectual Property Awareness and Attitudes (2022) <https://www.usipalliance.org/usipa-ip-attitudes-survey> accessed 14 July 2023.

122 ibid. See also: EUIPO, European Citizens and Intellectual Property: Perception, Awareness and Behaviour 2023 <https://euipo.europa.eu/tunnel-web/secure/webdav/guest/document_library/observatory/documents/reports/2023_IP_Perception_Study/2023_IP_Perception_Study_FullR_en.pdf> accessed 14 July 2023 which examines public awareness but does not refer to issues around knowledge of role of IPRs in access to health context.

123 This includes the following study of European Association for Endoscopic Surgery (EAES) members which found members had limited IPR awareness: Kiyokazu Nakajima and others, ‘The EAES Intellectual Property Awareness Survey’ (2022) 36 Surgical Endoscopy 36, 3340–46.

124 Jordan, Liddicoat and Liddell (n 113) 21–22 which discusses the (lack of) awareness of some biobanks of the potential clauses and options that could be adopted.

